# Characterisation of cultivation of the human cell line AGE1.HN.AAT

**DOI:** 10.1186/1753-6561-5-S8-P87

**Published:** 2011-11-22

**Authors:** Eva Schräder, Sebastian Scholz, Jens Niklas, Alexander Rath, Oscar Platas Barradas, Uwe Jandt, Volker Sandig, Thomas Rose, Ralf Pörtner, Udo Reichl, An-Ping Zeng, Elmar Heinzle, Thomas Noll

**Affiliations:** 1 Institute for Cell Culture Technology, University of Bielefeld, Germany; 2 Max Planck Institute for Dynamics of Complex Technical Systems, Magdeburg, Germany; 3 Institute for Bioprocess and Biosystems Engineering, Hamburg University of Technology, Germany; 4 Biochemical Engineering Institute, Saarland University, Saarbruecken, Germany; 5 ProBioGen AG, Berlin, Germany

## Background

Human cell lines are an interesting alternative to CHO cells for the production of recombinant proteins and monoclonal antibodies, because of their ability to produce genuine human posttranslational modifications. The human cell line AGE1.HN.AAT (ProBioGen, Berlin, Germany), that originated from human neural precursor tissue, has been adapted to serum-free conditions and cultivated in many different systems. Here we present our results using this cell line in a scale-up of batch cultivation from 50 mL vented polypropylene tube on a shaking platform, polycarbonate shakeflask (cultivation volume from 50 mL up to 300 mL), a 2 L-glass vessel stirred tank reactor and a 20 L-stainless steel stirred tank reactor (both Sartorius Stedim, Goettingen, Germany).

## Materials and methods

Cultivations were performed with chemically-defined and animal-component-free media 42-MAX-UB (Teutocell, Bielefeld, Germany). Batch-cultivations were performed in 50 mL-bioreactor tubes (TPP, Switzerland) shakeflasks (Corning Life Sciences, Netherlands), 2 L-glass vessel and 20 L-stainless steel vessel (both Sartorius-Stedim, Germany). Chemostat-cultivation was done in 0.5 L-bioreactor (DASGIP, Juelich, Germany) with a media exchange rate of 7 mL/h. As a further cultivation system a dialysis-reactor (Bioengineering, Wald, Switzerland) was established, with a 1.3 L cell-containing inner chamber and a 4 L media reservoir in the outer chamber, separated by a semipermeable dialysis membrane.

## Results

### Proliferation in controlled and uncontrolled systems

**Figure 1:**
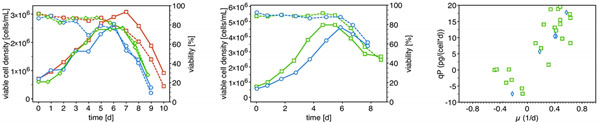
1.1 (left): viable cell density and viability during shake flask batch-cultivation. open squares: bioreactor tube, open circles: 125 mL-shakeflask, open diamonds: 250 mL-shakeflask. 1.2 (middle): viable cell density and viability during bioreactor batch-cultivation. open squares: 2 L-glass vessel, open circles: 20 L-stainless steel reactor. 1.3 (right): specific growth rate μ of bioreactor cultivation vs. corresponding specific productivity qP. open diamonds: 20 L- stainless steel reactor, open squares: 2 L-glass vessel

The AGE1.HN.AAT cells show similar growth in different unregulated vessels and culture volumes. The used systems ranged from 50 mL-bioreactor tubes with a culture volume of up to 18 mL, 125 mL-shakeflasks with a culture volume of up to 50 mL- to 250 mL-shakeflasks, in which a culture volume of 100 mL can be used (Results shown in figure 1a).

Cultivation in 2 L-glass vessel is as well feasible for batch process as well as preculture for 20 L-vessel. Cultivation at a 20 L-scale resulted in delayed cell growth but did not affect the final cell concentration (refer to figure 1b). AGE1.HN.AAT cells show a strong growth-coupled productivity as shown in figure 1c.

No difference between 2 L- and 20 L-vessel concerning spec. productivity and spec. growth rate were observed. A scale-up of cultivation-volume in batch process is definitely possible.

### Cultivations with other systems

Cultivation of AGE1.HN.AAT cells in dialysis-reactor is also possible and batch cultivation without media-exchange in the outer chamber showed maximum cell density up to 1.6 E7 viable cells per milliliter in the inner chamber after eight days of cultivation.

Chemostat-cultivation in 0.5 L-glass vessel shows constant cell density at 2.5 E6 cells/mL for more than 14 days.

## Conclusions

Cultivation of AGE1.HN cell line is possible in different regulated and unregulated systems and at different scales. Cell specific productivity and titer of the AGE1.HN.AAT producer cell line depend mainly on cell growth. The cells are easily scalable from 2 to 20 L batch cultivation in STR. Other cultivation strategies have been established successfully (incl. chemostat and dialysis-bioreactor) documenting the potential of the AGE1.HN cell line.

